# Does perception equal reality? Weight misperception in relation to weight-related attitudes and behaviors among overweight and obese US adults

**DOI:** 10.1186/1479-5868-8-20

**Published:** 2011-03-22

**Authors:** Dustin T Duncan, Kathleen Y Wolin, Melissa Scharoun-Lee, Eric L Ding, Erica T Warner, Gary G Bennett

**Affiliations:** 1 Department of Society, Human Development, and Health, Harvard School of Public Health, Boston, MA, USA; 2 Harvard Prevention Research Center on Nutrition and Physical Activity, Harvard School of Public Health, Boston, MA, USA; 3 Department of Surgery and Alvin J. Siteman Cancer Center, Washington University School of Medicine, St. Louis, MO, USA; 4 Duke Global Health Institute, Duke University, Durham, NC, USA; 5 Department of Nutrition, Harvard School of Public Health, Boston, MA USA; 6 Channing Laboratory, Department of Medicine, Brigham and Women's Hospital and Harvard Medical School, Boston, MA USA; 7 Department of Epidemiology, Harvard School of Public Health, Boston, MA, USA; 8 Center for Community-Based Research, Dana-Farber Cancer Institute, Boston, MA, USA; 9 Department of Psychology and Neuroscience, Duke University, Durham, NC, USA

## Abstract

**Background:**

Weight misperception might preclude the adoption of healthful weight-related attitudes and behaviors among overweight and obese individuals, yet limited research exists in this area. We examined associations between weight misperception and several weight-related attitudes and behaviors among a nationally representative sample of overweight and obese US adults.

**Methods:**

Data from the 2003-2006 National Health and Nutrition Examination Survey (NHANES) were used. Analyses included non-pregnant, overweight and obese (measured body mass index ≥ 25) adults aged 20 and older. Weight misperception was identified among those who reported themselves as "underweight" or "about the right weight". Outcome variables and sample sizes were: weight-loss attitudes/behaviors (wanting to weigh less and having tried to lose weight; n = 4,784); dietary intake (total energy intake; n = 4,894); and physical activity (meets 2008 US physical activity recommendations, insufficiently active, and sedentary; n = 5,401). Multivariable regression models were stratified by gender and race/ethnicity. Analyses were conducted in 2009-2010.

**Results:**

These overweight/obese men and women who misperceived their weight were 71% (RR 0.29, 95% CI 0.25-0.34) and 65% (RR 0.35, 95% CI 0.29-0.42) less likely to report that they want to lose weight and 60% (RR 0.40, 95% CI 0.30-0.52) and 56% (RR 0.44, 95% CI 0.32-0.59) less likely to have tried to lose weight within the past year, respectively, compared to those who accurately perceived themselves as overweight. Blacks were particularly less likely to have tried to lose weight. Weight misperception was not a significant predictor of total energy intake among most subgroups, but was associated with lower total energy intake among Hispanic women (change -252.72, 95% CI -433.25, -72.18). Men who misperceived their weight were less likely (RR 0.68, 95% CI 0.52-0.89) to be insufficiently active (the strongest results were among Black men) and women who misperceived their weight were less likely (RR 0.74, 95% CI 0.54, 1.00, *p *= 0.047) to meet activity recommendations compared to being sedentary.

**Conclusion:**

Overall, weight misperception among overweight and obese adults was associated with less likelihood of interest in or attempts at weight loss and less physical activity. These associations varied by gender and race/ethnicity. This study highlights the importance of focusing on inaccurate weight perceptions in targeted weight loss efforts.

## Background

At almost 70%, the prevalence of overweight and obesity continues to be at epidemic proportions in the US [1]. Given that overweight and obesity increase the risk for many leading chronic diseases (e.g. type 2 diabetes, hypertension, coronary heart disease, stroke, kidney disease, several types of cancer) [2] and premature morality [2,3], it remains one of today's most pressing public health and medical priorities.

*Misperception of weight status *(i.e. discordance between an individual's actual weight status and the perception of his/her weight status) has repeatedly been documented among overweight and obese adults [4-21]. It has been hypothesized that weight misperception among overweight and obese individuals may preclude the adoption of healthful attitudes and behaviors [4-11,13,15-17,19,22-27], perhaps as a result of lower weight loss motivation. Overweight and obese individuals who see themselves as healthy weight, for example, might not try to lose weight and might be less inclined to eat healthfully and be physically active. Surprisingly, however, little is known empirically about the influence of weight misperception on weight-related attitudes and behaviors among overweight and obese individuals; most studies on weight misperception usually only examine prevalence estimates of weight misperception by socio-demographic factors (e.g. gender and race/ethnicity). The limited available evidence indicates that inaccurate weight perceptions among overweight and obese individuals are associated with weight-related attitudes (such as eating concern and weight concern) [28] and weight-related behaviors (such as fewer weight loss attempts [29], unhealthful dietary intake [28-31] and lower physical activity levels) [29,30] -- key components inhibiting weight maintenance and loss. However, some evidence indicates that weight misperception among overweight and obese individuals might be associated with healthful behaviors (e.g. better diet quality [29], more physical activity [29], and less sedentary behavior [32].

Using data from the 2003-2006 National Health and Nutrition Examination Survey (NHANES), we sought to examine associations between weight misperception and several weight-related attitudes and behaviors (i.e. wanting to weigh less, having tried to lose weight, dietary intake, and physical activity) among overweight and obese US adults. We hypothesized that overweight and obese weight misperceivers would have unhealthful weight loss attitudes and behaviors (including decreased desire to weigh less, decreased attempts to have tried to lose weight, higher caloric intake and lower physical activity levels).

## Methods

### Study Design and Population

Data for this study come from the National Health and Nutrition Examination Survey (NHANES) conducted by the National Center for Health Statistics of the Centers for Disease Control and Prevention. NHANES is an on-going annual survey of health and nutritional status collected from a stratified, multi-stage probability sample of the civilian non-institutionalized US population, with an oversampling of targeted groups (including Blacks and Mexican-Americans) [33,34]. This study draws from two consecutive cycles of nationally representative data from 2003-2006, comprising 10,122 individuals interviewed in 2003-2004 and 10,348 individuals in 2005-2006. The study population was limited to non-pregnant, overweight and obese (measured body mass index [BMI] ≥ 25 using the formula: weight in kilograms divided by the square of height in meters; height and weight were measured under standard protocols) [35,36] adults aged 20 and older. To increase statistical power, we used a three-sample strategy in which the sample size was maximized in for each outcome type. Sample 1 was composed of overweight and obese adults who completed the weight loss attitudes/behaviors survey items (n = 4,784); sample 2 was composed of overweight and obese adults who completed the dietary intake survey items (n = 4,894); and sample 3 was composed of overweight and obese adults who completed the physical activity questionnaire (n = 5,401). This three-sample strategy helps us avoid any bias associated with conducting a complete case analysis.

### Weight Misperception

Respondents were asked if they considered themselves now to be "overweight, underweight, or about the right weight." Given the restriction of the sample to the overweight and obese, weight misperception (our predictor variable) was determined among those who reported themselves to be "underweight" or "about the right weight"; those who correctly perceived themselves as overweight served as the referent group.

### Weight-Related Attitudes and Behaviors

Three types of weight-related outcomes were examined: (1) weight loss attitudes/behaviors, (2) dietary intake and (3) physical activity. Weight loss attitudes/behaviors were captured using two binary variables: whether the respondent would like to weigh less (yes/weigh less or no/stay about the same), and whether he/she had tried to lose weight in the last year (yes/no). The dietary variable we examined was total energy intake (kcals). Energy intake were assessed as continuous variables using 24-hour recall data, averaging the values from 2 non-consecutive days of recall to capture usual intake (day 1 was administered in person during the physical examination, whereas day 2 was administered by phone a few weeks later). Physical activity was assessed using frequency (per month), duration (minutes) and intensity (metabolic equivalent [MET]) data on specific reported leisure-time activities for each individual. A 3-level variable was defined based on the 2008 U.S. Department of Health and Human Services' physical activity guidelines [37], where "meets activity recommendations" was defined as achieving 150 minutes/week of moderate intensity physical activity (3.0-5.9 METs) or 75 minutes/week of vigorous intensity physical activity (6.0+ METs) or an equivalent combination thereof (500 MET-min/week ≥ 3.0 METs). Those categorized as "insufficiently active" reported activity that did not exceed the above thresholds; respondents who did not report any leisure-time physical activity were categorized as "sedentary."

### Other Variables

Other variables used to define sample subgroups and/or as model covariates include: gender (male, female), race/ethnicity (non-Hispanic White, non-Hispanic Black, or Hispanic; other groups were excluded due to small sample sizes), age (years), annual household income (<$20,000, $20-35,000, $35-75,000 and $75,000+), education (<high school, high school graduate, some college, college graduate+), marital status (never married, widowed/divorced, married), self-rated health (fair/poor, good, very good/excellent), whether a physician or health professional had ever told the respondent that he/she was overweight (yes, no) as well as physical activity and BMI (both previously described).

### Statistical Analyses

Multivariable regression models were used to examine the association between weight misperception and each outcome. The form of regression was dependent on the distribution of the outcome variables. Binary and categorical variables were modeled using log-Poisson and multinomial logistic regression (respectively), whereas continuous variables were modeled via linear regression. Log-Poisson regression results for each binary outcome were exponentiated to a relative risk (RR). Relative risks (i.e., prevalence ratios) were calculated rather than odds ratios because the outcomes (i.e. wanting to weigh less and having tried to lose weight) are common among our study population of overweight and obese individuals; odds ratios are likely to overestimate the effect when the outcome is common [38-41]. Since the Poisson model specification may overinflate the standard error, we conducted sensitivity analysis using jacknife variance as the robust variance estimator to deflate them, however, the estimates and *p *values replicated were near identical as our initial standard approach (we therefore present findings from our initial approach). Multinomial logistic regression models for the 3-level physical activity outcome variable were used to estimate the likelihood of "meeting activity recommendations" or being "insufficiently active" relative to being "sedentary" (outcome referent) for those who misperceived their weight relative to those who correctly perceived their weight (exposure referent). We fit multinomial logistic regression models as opposed to ordinal logistic regression models because the intervals between the 3-level physical activity outcome variable were not equal and because initial exploratory data analysis revealed that the continuous physical activity variable was heavily skewed towards zero and the non-zero observations are continuous rather than ordinal, making other strategies inappropriate. Coefficients from these models were exponentiated to obtain estimates of the relative risk of each physical activity outcome as a function of weight misperception. Linear regressions produced beta coefficients estimating the change in the continuous total energy intake outcome based on weight misperception. In these models, we control for physical activity because because it is a predictor of dietary intake [42]. We also adjust for BMI in all models, given that misperception varies by actual BMI [11] and given that BMI influences the study outcomes [42-44]. All models were conducted for the entire sample as well as stratified by gender and race/ethnicity, since 1) we were interested in parameter estimates by gender and race/ethnicity, 2) there are gender and racial/ethnic differences in weight misperception among overweight/obese individuals [4-9,11,13,14,16-21,26] and 3) there are gender and racial/ethnic differences in weight-related attitudes and behaviors among overweight/obese individuals [8,45,46]. Statistical significance was determined by 95% confidence intervals (CIs) and *p *values less than 0.05. All analyses were conducted in 2009-2010 using STATA statistical software (version 10.0; Stata Corp, College Station, Texas), with survey procedures to correct for unequal probability of selection and underestimation of variance due to clustered sample design.

## Results

### Characteristics of the Study Population

Socio-demographic characteristics of the study population of overweight and obese US adults for the three outcomes are presented in Table 1. Slightly over half were overweight (50.1 to 51.5%). Approximately 23% misperceived their weight. More than half were male and about three-quarters of respondents were non-Hispanic White. The mean age was about 48 years. Most individuals (>80%) had high school education or greater and also earned over $20,000 annually. A little over 65% were currently married. The majority of these overweight and obese individuals reported "good" (34.1 to 35.2%), "very good" or "excellent" self-rated health (46.0 to 47.3%); only about 40% reported receiving a physician diagnosis of overweight. Additionally, most of these overweight and obese individuals (80.6%) wanted to lose weight and 47.4% had tried to lose weight in the past year. The mean total energy intake of the study population was 2138.5 kcals (SE: 22.2). About one-third (33.8%) were sedentary, 25.2% were insufficiently activity and 41.0% reported meeting physical activity recommendations.

**Table 1:** Characteristics of Overweight and Obese U.S. adults aged ≥ 20, NHANES 2003-2006

	Weight Loss Attitudes and Behaviors Sample % (n)	Dietary Behaviors Sample % (n)	Physical Activity Behaviors Sample % (n)
Total	n = 4,784	n = 4,894	n = 5,401

Weight Misperception (%)	23.7 (1389)	22.9 (1296)	22.5 (1503)

Weight Status			

Overweight	51.5 (2487)	50.2 (2442)	50.1 (2729)

Obese	48.5 (2297)	49.8 (2452)	49.9 (2672)

BMI (kg/m^2^, mean, SE)	31.3 (0.13)	31.4 (0.16)	31.4 (0.12)

Age (years, mean, SE)	48.2 (0.52)	48.5 (0.51)	47.7 (0.50)

Gender			

Men	53.4 (2537)	52.2 (2543)	53.1 (2866)

Women	46.6 (2247)	47.8 (2351)	46.9 (2535)

Race/Ethnicity			

Non-Hispanic White	73.9 (2426)	75.1 (2562)	74.1 (2737)

Non-Hispanic Black	13.4 (1161)	13.3 (1155)	13.4 (1331)

Hispanic	12.8 (1197)	11.7 (1177)	12.5 (1333)

Education			

High School or Less	18.1 (1402)	16.4 (1114)	17.2 (1508)

High School Graduate	27.8 (1233)	19.4 (1141)	27.7 (1387)

Some College	32.3 (1343)	35.4 (1595)	32.6 (1564)

College Graduate	21.7 (806)	28.8 (1044)	22.5 (942)

Income			

$<20,000	16.9 (1170)	16.6 (1340)	15.8 (1250)

$20,000-35,000	19.0 (1099)	28.0 (1267)	18.8 (1230)

$35,000-75,000	36.7 (1553)	32.9 (1425)	36.8 (1780)

$>75,000	27.5 (962)	22.6 (862)	28.7 (1141)

Marital Status			

Married	67.4 (2998)	66.2 (3119)	67.2 (3389)

Widowed/Divorced	19.4 (1125)	19.8 (1127)	18.9 (1223)

Never Married	13.3 (661)	14.0 (648)	13.9 (789)

Self-reported Health Status			

Fair or Poor	18.7 (1205)	18.3 (1194)	17.8 (1290)

Good	35.2 (1709)	34.1 (1737)	34.9 (1918)

Excellent or Very Good	46.0 (1870)	47.6 (1963)	47.3 (2193)

Physician Diagnosis for Overweight (%)	40.0 (1869)	43.8 (2130)	43.5 (2282)

*Note*. Weighted proportions are shown. These weighted data adjust for unequal probabilities of selection. Percentages may not total 100 due to rounding. Totals may not sum to final sample size due to missing data.

### Effect of Weight Misperception on Weight Loss Attitudes and Behaviors

Weight misperception was a strong predictor of weight loss attitudes and behaviors for both genders and all racial/ethnic groups (Table 2). Men and women who misperceived their weight were 71% (RR 0.29, 95% CI 0.25-0.34) and 65% (RR 0.35, 95% CI 0.29-0.42) less likely, respectively, to report wanting to lose weight than those who accurately perceived themselves as overweight. Similarly, men and women who misperceived their overweight status were 60% (RR 0.40, 95% CI 0.30-0.52) and 56% (RR 0.44, 95% CI 0.32-0.59) less likely than those who accurately perceived their overweight status to have attempted weight loss during the past year. Weight misperception was a particularly strong predictor among Blacks. Black men and women who misperceived their weight were 77% less likely to have tried to lose weight in the past year compared to Blacks who accurately perceived themselves as overweight. Based on within-group comparisons, this compares to a 62% reduction among Hispanic men, a 33% reduction among Hispanic women, a 55% reduction among White men, and a 56% reduction among White women.

**Table 2:** Effect of Weight Misperception on Weight Loss Attitudes and Behaviors Among Overweight and Obese US Adults by Gender and Race/Ethnicity, NHANES 2003-2006^a^

		Weight Loss Attitudes and Behaviors	
		**Wants To Lose Weight RR (95% CI)**	**Has Tried To Lose Weight ** **RR (95% CI)**

Total	Total	**0.30 (0.26, 0.35)**	**0.40 (0.33, 0.47)**
	
	Men	**0.29 (0.25, 0.34)**	**0.40 (0.30, 0.52)**
	
	Women	**0.35 (0.29, 0.42)**	**0.44 (0.32, 0.59)**

White	Men	**0.33 (0.27, 0.39)**	**0.45 (0.33, 0.62)**
	
	Women	**0.40 (0.32, 0.51)**	**0.44 (0.29, 0.67)**

Black	Men	**0.17 (0.12, 0.25)**	**0.23 (0.15, 0.35)**
	
	Women	**0.21 (0.13, 0.33)**	**0.23 (0.11, 0.47)**

Hispanic	Men	**0.23 (0.16, 0.32)**	**0.31 (0.20, 0.49)**
	
	Women	**0.38 (0.27, 0.53)**	**0.67 (0.53, 0.86)**

*Note*. RR = Relative Risk. CI = Confidence Interval. Reference Category = Adults who did not misperceive their weight status. Total columns are gender-adjusted.

^a ^Multivariable log-Poisson regression models were adjusted for body mass index, age, education, income, marital status, self-reported health status, and receipt of medical diagnosis of overweight.

### Effect of Weight Misperception on Dietary Intake and Physical Activity

Weight misperception was not a significant predictor of total energy intake among most subgroups (Table 3). Among Hispanic women, however, weight misperception was associated with lower total energy intake (change -252.72, 95% CI -433.25, -72.18). Weight misperception was a predictor of physical activity among certain gender and racial/ethnic groups. As compared to men who accurately perceive themselves as overweight, men who misperceived their weight were 32% less likely (RR 0.68, 95% CI 0.52-0.89) to be insufficiently active and as likely to meet activity recommendations as compared to being sedentary. The strongest results were among Black men. Black men who misperceived their weight as compared to Black men who accurately perceived their weight were 51% less likely (RR 0.49, CI 0.26-0.90) to be insufficiently active and as likely to meet activity recommendations compared to being sedentary; this compares to a reduction of 30% among White men (RR 0.70, 95% CI 0.50-1.00, *p *= 0.049). Weight misperception was not a significant predictor of insufficient activity among women of any racial/ethnic group and there was no significant effect of weight misperception on the odds of meeting activity recommendations compared to being sedentary among men. However, women who misperceived their weight as compared to women who accurately perceived their weight were 26% less likely (RR 0.74, 95% CI 0.54, 1.00, *p *= 0.047) to meet activity recommendations compared to being sedentary. This means that several groups of weight misperceivers were less physically active than those who accurately perceived their weight.

**Table 3:** Effect of Weight Misperception on Dietary Intake and Physical Activity Behaviors Among Overweight and Obese US Adults by Gender and Race/Ethnicity, NHANES 2003-2006^a^

		**Dietary Intake**^b^	**Physical Activity Behaviors**^c^	
		**Total Energy ** **Intake (kcals) ** **Change (95% CI)**	**Insufficiently ** **Active ** **RR (95% CI)**	**Meets Activity ** **Recommendations ** **RR (95% CI)**

Total	Total	9.53 (-85.24, 104.32)	**0.74 (0.60, 0.92)**	0.86 (0.68, 1.10)
	
	Men	10.54 (-107.68, 128.76)	**0.68 (0.52, 0.89)**	0.95 (0.69, 1.30)
	
	Women	-64.29 (-161.26, 32.69)	0.79 (0.55, 1.14)	**0.74 (0.54, 1.00)**

White	Men	7.02 (-139.73, 153.76)	**0.70 (0.50, 1.00)**	0.94 (0.61, 1.43)
	
	Women	-53.73 (-169.19, 61.74)	0.86 (0.53, 1.40)	0.75 (0.49, 1.13)

Black	Men	36.08 (-162.43, 234.60)	**0.49 (0.26, 0.90)**	1.01 (0.63, 1.61)
	
	Women	13.19 (-296.24, 322.62)	0.65 (0.37, 1.13)	0.55 (0.23, 1.35)

Hispanic	Men	55.46 (-189.94, 300.86)	0.68 (0.40, 1.18)	1.07 (0.67, 1.71)
	
	Women	**-252.72 (-433.25, -72.18)**	0.61 (0.29, 1.27)	1.10 (0.56, 2.15)

*Note*. Change = Beta Coefficient. RR = Relative Risk. CI = Confidence Interval. Exposure Reference Category = Adults who did not misperceive their weight status. The multinomial logistic regression outcome reference group is sedentary adults; Total columns are gender-adjusted.

^a ^Multivariable regression models were adjusted for body mass index, age, education, income, marital status, self-reported health status, and receipt of medical diagnosis of overweight.

^b ^We also adjusted for physical activity in the total energy intake models.

^c ^Physical activity recommendations issued by the U.S. Department of Health and Human Services for 2008, defined as achieving greater than or equal to 150 minutes of moderate or 75 minutes of vigorous intensity physical activity per week, or a combination thereof. Those categorized as insufficiently active reported activity that did not exceed the above thresholds while those who did not report any physical activity were categorized as sedentary.

## Discussion

Weight misperception among overweight and obese populations is of public health and medical significance and may limit the effectiveness of weight loss and obesity prevention efforts. Nearly one-quarter of our representative US sample of overweight and obese adults misperceived their weight status. In this study, overweight and obese individuals who misperceived their weight status were less likely to want to lose weight and having tried to lose weight as compared to overweight and obese individuals who accurately perceived their weight. This effect was apparent among both men and women and among all racial/ethnic groups, but was especially pronounced for Black men and women. Weight misperception was not a significant predictor of dietary behaviors for most subgroups, but was associated with lower total energy intake among Hispanic women. Additionally, men (especially Black men) who misperceived their weight as compared those who accurately perceived their weight were less likely to be insufficiently active compared to being sedentary and women who misperceived their weight as compared to women who accurately perceived their weight were less likely to meet activity recommendations compared to being sedentary. Importantly, this is the first study, to the best of our knowledge, to have examined weight misperception in relation to a variety of weight-related attitudes and behaviors among a nationally representative sample of overweight and obese men and women in the US.

Findings from our study are largely consistent with the few existing studies in this area. Jones et al., for example, found that adults with class II obesity (BMI = 35.0-39.9) who had inaccurate weight perceptions had less weight concern, less distress regarding overeating, less distress regarding control overeating, less emotional overeating, less eating disinhibition as well as exhibited a nonsignificant trend toward less time spent dieting [28]. Forman et al. found that overweight adults who misperceived themselves as average weight were dieting less often than those who correctly perceived their weight status [31]. In a study of overweight adolescents with type 2 diabetes, Skinner et al. found that those who misperceived their weight had poorer diet such as higher consumption of sugary drinks, eating fast food, having unplanned snacks, and overeating along with low levels of physical activity and more sedentary time [30]. Edwards et al. found that overweight adolescents in the Youth Risk Behavior Survey who accurately perceived their weight were more likely to report trying to lose weight as well as more likely to exercise and consume fewer calories in the past 30 days, but overweight boys who accurately perceived their weight were also less likely to report achieving recommended levels of fruit/vegetable intake and physical activity in the previous week [29]. Taken together, these findings suggest the importance of considering weight perceptions in the design of future behavioral weight loss interventions.

Theories of health behavior provide a useful lens to interpret these findings. Several widely used theories such as the Health Belief Model [47] suggest that perceived susceptibility to a given condition is necessary to promote healthful behavior change. Consistent with health behavior theory, our findings show that misperceivers are less likely to plan or attempt weight loss, and more likely to overall perform behaviors that increase their likelihood of experiencing weight gain. One notable caveat is our finding that Hispanic women who misperceived their weight status had lower energy intake than those who correctly perceive their weight. However, our overall findings and those of others by and large [28-31] suggest that correcting perception of weight status may be an important consideration in the design of weight loss interventions, particularly those conducted among high-risk subgroups. Intervention efforts are especially needed for overweight and obese men and Black adults, given the groups' high prevalence of weight misperception [4-9,11,13,14,16-21,26], their elevated rates of overweight and obesity [1], and their consistent and strong associations of weight misperception in relation to unhealthful weight-related attitudes and behaviors seen in the present study. We also note that the misperception that should cause the greatest concern is that of extremely obese individuals (almost 50% of our representative sample is obese) for whom, independent of racial/ethnic group, elevated health risks are most certain [48,49].

Weight misperceptions are potentially modifiable. There are several possible strategies for counteracting misperceptions in the primary care setting and in line with these findings only about 40% of the overweight and obese respondents in our sample reported being told by a physician or health professional that they were overweight. However, when provider counseling occurs, it can be particularly helpful. Several studies have shown that when clinicians advise their obese patients to lose weight, there is an increased likelihood of weight loss attempts [50,51]. Additionally, the clothing industry might be encouraged to revisit shared clothing sizing standards. The elimination of "vanity sizing" (also known as size inflation, whereby clothing size numbers scale down over time; e.g. a size 14 becomes a size 10) [52] might reduce weight misperception -- as some individuals monitor their weight with clothing sizes [53]. Marketing campaigns changing societal norms that encourage weight misperception also could be implemented. Such norms exist among men (e.g. overweight men have greater body image satisfaction [19,54,55] and men value heavier body weight) [18,21,26,27,56] and certain racial/ethnic minority groups (e.g. Blacks have greater body image satisfaction independent of their body weight [57-61] and maintain a greater social acceptance of heavier body weight) [61-65]. However, it is important to note that such intervention efforts should be carefully crafted to protect against eating disorders, body image disorders and emotional distress, as these responses may be experienced when weight misperceptions are corrected.

We note that this study also is subject to some limitations. First, many of the gender- and racial/ethnic-stratified models yielded wide confidence intervals and null associations (e.g. all models estimating total energy intake had very wide confidence intervals and we speculate the effect estimates from the energy intake models are unstable); although we maximized the sample size for each analysis for increased power and to avoid any bias by not doing so, lack of power due to smaller sample sizes in these cells might be implicated in the findings. Second, the cross-sectional design of this study does not allow causal conclusions to be drawn. However, despite the well-known limitations of cross-sectional data, our study hypotheses and directionality have intuitive appeal and were based on conclusions from past theoretical and empirical research. Additionally, we relied on self-reported data on health behaviors (i.e. diet and physical activity). Although there can be reliability and validity challenges with self-reported dietary and physical activity measures [66-69] (which may be especially problematic among overweight and obese individuals), this type of data is most commonly used in population-based health research. We report only on leisure-time physical activity, which is only one domain of physical activity behavior and may vary by socio-demographic characteristics, especially race/ethnicity [70,71]. BMI was used in this study, as is commonly done in population-based research. Nevertheless, previous research has noted that BMI is an imperfect measure of body composition that does not take into account body fat distribution or body fatness (e.g. the ratio of muscle to fat) [72], which overweight and obese individuals are likely to take into consideration when determining their own weight status [14] and it may vary by gender and race/ethnicity. The possibility of temporal differences in the time since receipt of diagnosis exists because the medical diagnosis of overweight was not time-delimited (e.g. prior 12 months). Furthermore, as with all observational studies, the possibility of residual confounding cannot be eliminated. However, we adjusted for multiple potential confounding variables in this study. Lastly, Mexicans were largely overrepresented in NHANES and therefore these results might not be generalizeable to other Hispanic subgroups.

The outcomes we selected for this study are reflective of attitudes and behaviors that are necessary for successful weight loss. As such, to enhance the interpretability of our findings, we chose behaviors that are most directly related to weight regulation: total calories and energy expenditure (however other behavioral aspects might be relevant to weight misperception). There is a need for additional research to replicate, extend and contextualize our findings. As the few studies examining weight misperception in relation to weight-related attitudes and behaviors were cross-sectional, longitudinal study designs are needed to establish the temporal ordering of study variables. It is important to note that weight misperception can be examined in multiple ways [22]. In our study, furthermore we do not know what respondents were using as a reference point when reporting weight status. Respondents might have compared themselves with their personal standards of a desired size (which might be based on cultural ideals and/or the size of their family or friends), medical standards of a certain weight for height (e.g. statements from a physician) or some other standard. Finally, in order to correct weight misperception, research is needed to examine causes of weight misperception among overweight and obese individuals (which have yet to be fully elucidated). Social comparison might be an explanation for weight misperception. Research indicates that being exposed to obesity is associated with greater weight misperception (underestimation) [24] and that increased obesity prevalence rates over the years has been associated with fewer overweight individuals perceiving themselves as overweight [5,73,74], increased body weight norms [4,73], and increased desired and ideal weights [25,75]. Additionally, we note that weight misperceptions might differ between groups because some experience a weaker BMI-mortality gradient than others (e.g. obesity--until the extreme range--is less lethal for Blacks as compared to Whites) [48].

## Conclusion

Overall, we found that weight misperception among overweight and obese adults was associated with less likelihood of interest in or attempts at weight loss and less physical activity. These associations varied by gender and race/ethnicity. This study highlights the importance of focusing on inaccurate weight perceptions in targeted weight loss efforts among overweight and obese individuals.

## Competing interests

The authors declare that they have no competing interests.

## Authors' contributions

DTD led the conceptualization and implementation of the analysis plan, directed the statistical analysis, interpreted the results as well as drafted and revised the manuscript. KYW conceived the analysis and study hypothesis, contributed to the analysis plan, contributed to data interpretation and critically revised the manuscript for substantial intellectual content. MSL contributed to the analysis plan, conducted the statistical analysis, contributed to data interpretation and drafted the initial methods section. ELD assisted in conducting the statistical analysis and contributed to data interpretation. ETW contributed to the analysis plan, interpreted the results and drafted the initial results section. GGB conceived the analysis and study hypothesis, contributed to the analysis plan, contributed to data interpretation and critically revised the manuscript for substantial intellectual content. All authors read and approved the final manuscript.
